# CBFA2T3 Is PPARA Sensitive and Attenuates Fasting-Induced Lipid Accumulation in Mouse Liver

**DOI:** 10.3390/cells13100831

**Published:** 2024-05-13

**Authors:** Donghwan Kim, Sang Keun Ha, Frank J. Gonzalez

**Affiliations:** 1 Division of Functional Food Research, Korea Food Research Institute, Wanju-gun 55365, Republic of Korea; skha@kfri.re.kr; 2 Center for Cancer Research, National Cancer Institute, National Institutes of Health, Bethesda, MD 20892, USA; gonzalef@mail.nih.gov; 3 Division of Food Biotechnology, University of Science and Technology, Daejeon 34113, Republic of Korea

**Keywords:** Cbfa2t3, Mtg16, PPARA, liver, fasting, Car5a, Hspa1b

## Abstract

Peroxisome proliferator-activated receptor alpha (PPARA) is a ligand-activated transcription factor that is a key mediator of lipid metabolism and metabolic stress in the liver. Accumulating evidence shows that PPARA regulates the expression of various protein coding and non-coding genes that modulate metabolic stress in the liver. CBFA2/RUNX1 partner transcriptional co-repressor 3 (CBFA2T3) is a DNA-binding transcription factor that belongs to the myeloid translocation gene family. Many studies have shown that CBFA2T3 is associated with acute myeloid leukemia. Especially, CBFA2T3–GLIS2 fusion is a chimeric oncogene associated with a poor survival rate in pediatric acute megakaryocytic leukemia. A previous study identified that PPARA activation promoted *Cbfa2t3* induction in liver and that *Cbfa2t3* may have a modulatory role in metabolic stress. However, the effect of CBFA2T3 gene expression on metabolic stress is not understood. In this study, the PPARA ligand WY14643 activated *Cbfa2t3* expression in mouse liver. Glucose tolerance test and insulin tolerance test data showed that insulin resistance is increased in *Cbfa2t3*^−/−^ mice compared to *Cbfa2t3*^+/+^ mice. Hepatic CBFA2T3 modulates heat shock protein family A member 1b and carbonic anhydrase 5a expression. Histology analysis revealed lipid droplet and lipid accumulation in the liver of fasting *Cbfa2t3*^−/−^ mice but not *Cbfa2t3*^+/+^ mice. The expression of lipid accumulation-related genes, such as *Cd36*, *Cidea*, and *Fabp1*, was increased in the liver of fasting *Cbfa2t3*^−/−^ mice. Especially, basal expression levels of *Cidea* mRNA were elevated in the liver of *Cbfa2t3*^−/−^ mice compared to *Cbfa2t3*^+/+^ mice. Much higher induction of *Cidea* mRNA was seen in the liver of *Cbfa2t3*^−/−^ mice after WY14643 administration. These results indicate that hepatic CBFA2T3 is a PPARA-sensitive gene that may modulate metabolic stress in mouse liver.

## 1. Introduction

CBFA2/RUNX1 partner transcriptional corepressor 3 (CBFA2T3) belongs to the CBFA family, which consists of CBFA2T1 (also known as RUNX1T1), CBFA2T2, and CBFA2T3 [1]. CBFA2T3, also known as MTG16 or ETO2, belongs to the myeloid translocation gene family, which binds to DNA-bound transcription factors and recruits corepressors to facilitate transcriptional repression [2]. *Cbfa2t3*^−/−^ mice have no anatomical or developmental abnormalities suggesting possible functional redundancy between CBFA2T3 family members [3]. However, CBFA2T3 is important for the regulation of hematopoietic stem/progenitor cell proliferation and differentiation [2]. CBFA2T3 expression levels are elevated in lymphoid and myeloid based cell lines, and genetic CBFA2T3 fusion with RUNX1 or GLIS2 accelerates acute myeloid leukemia and megakaryoblastic leukemia [4]. Especially, CBFA2T3–GLIS2 fusion is a most common phenotype in pediatric (not adult) acute myeloid leukemia that is associated with a poor clinical outcome [5]. Therefore, many studies about AML in pediatrics have focused on the CBFA2T3–GLIS2 fusion [6].

*Cbfa2t3*^−/−^ mice have a lower number of B-lymphocytes and megakaryocytic erythroid progenitors concurrent with an increased proportion of granulocytic monocytic progenitors compared with *Cbfa2t3*^+/+^ mice [2]. A recent study showed that CBFA2T3 is a key factor for cell fate decisions under colonic homeostasis, which modulates colitis and colon tumorigenesis by inhibiting E protein transcription factors [7]. CBFA2T3 suppresses hypoxia-inducible factor 1 alpha (HIF1A) through proteasome pathway activation by directly interacting with HIF1A in B-lymphoblastic Raji cells [8]. CBFA2T3 gene expression is elevated in lung adenocarcinoma cell lines A549 and H2122, and CBFA2T3 has been identified as potential tumor antigens for mRNA vaccine development in lung adenocarcinoma [9,10]. CBFA2T3 overexpression suppresses MED19 levels and inhibits breast cancer cell proliferation in MCF-7 and MDA-MB-231 cells [11].

Peroxisome proliferation activated receptor α (PPARA) is a key mediator of hepatic lipid metabolism through activation of genes involved in lipid catabolism and transport [12]. PPARA activation modulates fatty acid oxidation and protects the liver from fasting-induced metabolic stress [13]. WY14632 is an exogenous PPARA ligand that modulates inflammation, lipid metabolism, cell proliferation and signaling pathways [14]. RNA sequencing analysis from the liver of PPARA ligand WY14643 treated mice revealed many potential PPARA target genes transcribed in response to PPARA activation [15]. Highly liver specific PPARA target long non-coding RNA GM15441 and G23RIK expression were elevated by PPARA activation, which modulated the metabolic stress induced by WY14643 and fasting in liver [16,17]. Especially, PPARA activation suppressed fasting-induced metabolic stress through its target gene activation [13,16,17].

Interestingly, *Cbfa2t3* expression was elevated by PPARA activation by WY14643 administration and *Cbfa2t3* may play a role in metabolic remodeling [16]. However, the modulation of metabolic stress by CBFA2T3 is unknown. In the present study, the basal expression level of hepatic CBFA2T3 was low but highly induced by WY14643; this induction did not occur in *Ppara*^−/−^ mice. In wild-type mice, PPARA induced mRNAs encoding heat shock protein family A member 1B (HSPA1B) and carbonic anhydrase 5a (CAR5A) mRNA expression while *Hspa1b* and *Car5a* mRNA expression remained at a control state in *Cbfa2t3*^−/−^ mice. Fasting significantly induced hepatic *Cbfa2t3* mRNA expression and this was not observed in *Ppara^−/−^* mice. Lipid accumulation was increased in *Cbfa2t3*^−/−^ mice compared to *Cbfa2t3*^+/+^ mice along with lipid accumulation-related gene activation such as cell death-inducing DNA fragmentation factor alpha subunit-like effector A (*Cidea*), cluster of differentiation 36 (*Cd36*) and fatty acid binding protein 1 (*Fabp1*) mRNAs. Especially, the basal expression level of *Cidea* mRNA was elevated in the liver of *Cbfa2t3*^−/−^ mice compared to *Cbfa2t3*^+/+^ mice, and the induction of *Cidea* mRNA expression in the liver by WY14643 administration was highly elevated in *Cbfa2t3*^−/−^ mice compared to *Cbfa2t3*^+/+^ mice. CBFA2T3 is a PPARA-sensitive gene that modulates metabolic stress in mouse liver.

## 2. Materials and Methods

### 2.1. In Vivo Models

Male 8-to-12-weeks old mice on the C57BL/6J background were used for all studies. *Ppara*^+/+^ male mice and conventional *Ppara*^−/−^ male mice used in this study were previously reported [18]. For WY14643 administration, the mice were supplied a NIH-31 diet or matched WY14643-containing NIH-31 diet (0.1%) for 2 days. For monitoring the gene response to PPARA activation in liver, WY14643 (50 mg/kg in 200 µL) dissolved in 1% carboxymethyl cellulose (CMC) was administered by gavage at the indicated time points. At the indicated time points, mice were euthanized by CO_2_ asphyxiation and then their tissues were harvested. The *Cbfa2t3*^+/+^ male mice and *Cbfa2t3*^−/−^ male mice used in this study were reported previously [2]. Mice were housed in light (12 h light: 12 h darkness cycle) and temperature-controlled rooms (humidity 40–60%) and were provided with water and pelleted chow ad libitum. Mice were euthanized by CO_2_ and tissue samples were harvested. Blood was collected by venipuncture of the caudal vena cava. All animal experiments were performed in accordance with Assessment and Accreditation of Laboratory Animal Care International Guidelines Association and approved by the National Cancer Institute Animal Care and Use Committee (LM-085).

### 2.2. Blood Serum Biochemistry

BD Microtainer Serum Separator Tubes were used to collect blood from mice (Becton Dickinson, Franklin Lakes, NJ, USA). Blood serum analysis for total cholesterol (CHOL), triglycerides (TG), and non-esterified fatty acids (NEFA) was performed using Wako Clinical Diagnostics kits (WakoUSA, Richmond, VA, USA). Catachem VETSPEC Kits were used to analyze serum alanine aminotransferase (ALT) and aspartate aminotransferase (AST) levels according to the manufacturer’s recommended instructions (Catachem, Oxford, CT, USA).

### 2.3. Histological Analysis

Fresh liver tissue was fixed in 10% phosphate-buffered formalin for 1 day and then processed in paraffin blocks. Sections of 4 μm were used for hematoxylin and eosin (H&E) staining. For Oil Red O (ORO) staining, fresh liver pieces were placed in a standard cryomold and covered with OCT Compound (Tissue-Tek, Sakura Finetek, Torrance, CA, USA), then stored at −80 °C. Sectioning and staining were performed by HistoServ, Inc. (Germantown, MD, USA). Slide imaging was analyzed using a Keyence BZ-X700 microscope (Keyence, Osaka, Japan) with 20× objectives, 200× magnification. The quantified lipid droplet area was analyzed using ImageJ software 1.54g (NIH, Bethesda, MD, USA).

### 2.4. Gene Expression Analysis by qRT-PCR

Fresh liver tissues were homogenized and total RNA extracted using TRIzol (Thermo Fisher Scientific, Waltham, MA, USA). Total RNA was quantified using a NanoDrop Spectrophotometer (NanoDrop Products, Wilmington, DE, USA), then 2 μg of RNA was reverse transcribed by All-in-One cDNA Synthesis SuperMix (BioTool, Houston, TX, USA). SYBR Green qPCR Master Mix (BioTool) was used for the qRT-PCR analysis. Primer-BLAST was used to design specific mRNA primers (www.ncbi.nlm.nih.gov/tools/primer-blast/) and they were purchased from IDT DNA Technologies (Coralville, IA, USA) (Table 1). The qRT-PCR results were normalized to *Gapdh*. Values given are fold over control or relative expression value, where appropriate, calculated using the 2^−ΔΔCt^ qRT-PCR calculation method [19].

### 2.5. Metabolic Analysis

For the glucose tolerance tests (GTT), mice were fasted for 16 h. For the insulin tolerance tests (ITT), the mice were fasted for 4 h. Glucose at 2 g/kg or insulin (Eli Lilly, Washington, DC, USA) at 0.8 U/kg in saline were intraperitoneally injected and blood glucose measured from tail bleeds using a Contour Glucometer (Bayer, Mishawaka, IN, USA). Blood glucose was measured before the injection and at intervals of 15 min up to 2 h post injection using the glucometer.

### 2.6. Statistical Analysis

All data are expressed as mean ± standard deviation. Significance was tested by *t*-tests using Prism 10.0 software (GraphPad Software, La Jolla, CA, USA). Differences were considered statistically significant at a *p* value less than 0.05. * *p* < 0.05; ** *p* < 0.01; *** *p* < 0.001.

## 3. Results

### 3.1. Hepatic Cbfa2t3 Is Induced by PPARA

**Figure 1:**
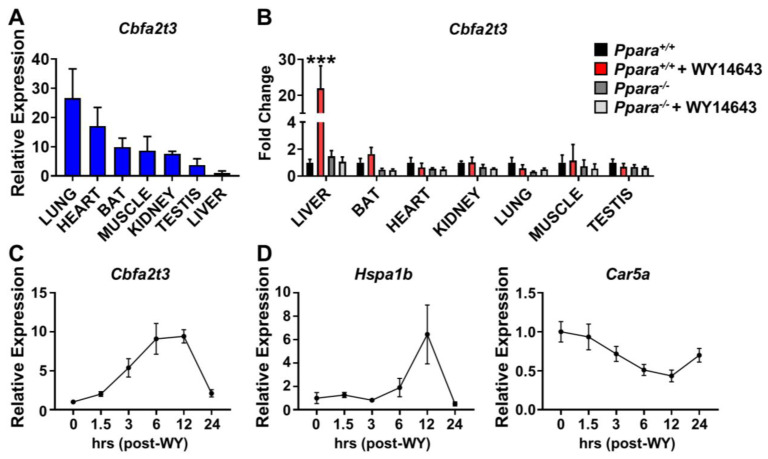
PPARA induces hepatic *Cbfa2t3*. WY14643 was administered to *Ppara*^+/+^ and *Ppara*^−/−^ mice for 2 days. (**A**) Relative basal *Cbfa2t3* mRNA expression in tissues. (**B**) *Cbfa2t3* mRNA expression in mice with and without WY14643. (**C**) Time course for changes in expression in liver of *Cbfa2t3* mRNA following WY14643 administration. (**D**) Time course for changes in expression in liver of *Hspa1b* and *Car5a* mRNAs following WY14643 treatment. Each data point represents the mean ± SD for *n* = 5. *** *p* < 0.001 vs. *Ppara*^+/+^.

Basal expression of *Cbfa2t3* mRNA was most highly elevated in lung, heart, and brown adipose tissue (BAT) (Figure 1A). To verify the effect of PPARA activation on *Cbfa2t3* expression and its tissue specificity, *Ppara*^+/+^ and *Ppara*^−/−^ mice were given WY14643 for 2 days, and seven tissues were harvested. *Cbfa2t3* mRNA was significantly induced in the liver by WY14643 while no induction was found in *Ppara*^−/−^ mice (Figure 1B). No significant activation was identified in the six other tissues, suggesting that *Cbfa2t3* expression is consistent with the tissue specificity of PPARA expression and activity (Figure 1B).

To monitor the *Cbfa2t3* gene response to PPARA activation, a single dose of WY14643 was given to mice by gavage. In the liver, *Cbfa2t3* induction was rapidly detected at 1.5 h and maximum expression of *Cbfa2t3* mRNA was identified 12 h after WY14632 administration (Figure 1C). RNA-seq analysis showed that heat shock protein family A member 1b (*Hspa1b*) and carbonic anhydrase 5a (*Car5a*) transcript levels were modulated by PPARA activation [16]. qRT-PCR results showed that PPARA also activated the expression of *Hspa1b* mRNA and repressed *Car5a* mRNA in liver (Figure 1D). These data indicate that hepatic *Cbfa2t3* is a PPARA-sensitive gene, indicating that *Cbfa2t3* may play a role in metabolic remodeling.

### 3.2. Cbfa2t3 Modulates Glucose Metabolism and Insulin Sensitivity

PPARA is a major regulator of lipid homeostasis and its target genes encode enzymes and transporters that have a critical regulatory role in controlling lipid and glucose homeostasis [16,17]. Previous data showed that *Cbfa2t3* induction was seen after PPARA activation, suggesting *Cbfa2t3* may have a modulatory role on metabolic stress in liver (Figure 1B,C).

**Figure 2:**
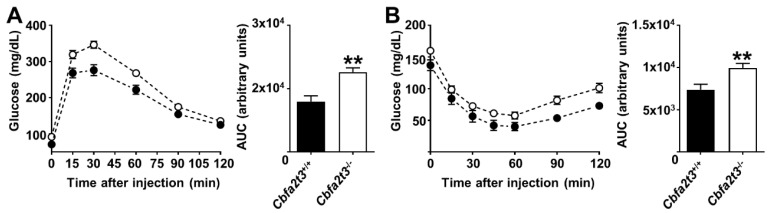
*Cbfa2t3* modulates glucose metabolism and insulin sensitivity. (**A**) GTT in *Cbfa2t3*^+/+^ and *Cbfa2t3*^−/−^ mice and calculated area under the curve (AUC) for GTT tests (*n* = 5). (**B**) ITT in *Cbfa2t3*^+/+^ and *Cbfa2t3*^−/−^ mice and calculated AUC for ITT tests (*n* = 5). Each data point represents the mean ± SD for *n* = 5. ** *p* < 0.01 vs. *Cbfa2t3*^+/+^.

To explore the role of *Cbfa2t3* in glucose metabolism and insulin sensitivity, GTT and ITT were performed in *Cbfa2t3*^+/+^ and *Cbfa2t3*^−/−^ mice. GTT results showed that blood glucose levels were increased in *Cbfa2t3*^−/−^ mice compared to *Cbfa2t3*^+/+^ mice (Figure 2A). ITT results also showed that blood glucose levels were increased after injection of 0.8 U/kg insulin, which was clearly marked in *Cbfa2t3*^−/−^ mice compared to *Cbfa2t3*^+/+^ mice (Figure 2B). These data suggest that *Cbfa2t3* modulates metabolic stress (Figure 2A,B).

### 3.3. Impact of Cbfa2t3 Deficiency in the Liver

**Figure 3:**
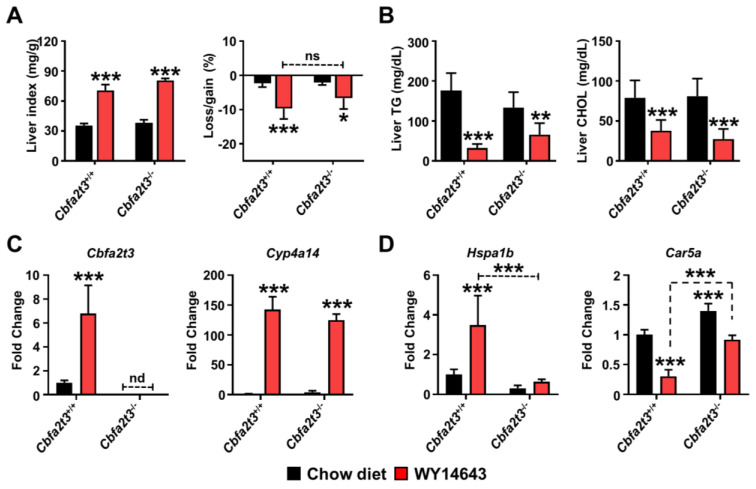
Impact of *Cbfa2t3* deficiency in the liver. WY14643 administered to *Cbfa2t3*^+/+^ and *Cbfa2t3*^−/−^ mice for 2 days. (**A**) Liver index (mg liver/g total body mass) and body weight loss in *Cbfa2t3*^+/+^ and *Cbfa2t3*^−/−^ mice. (**B**) TG and CHOL levels in *Cbfa2t3*^+/+^ and *Cbfa2t3*^−/−^ mice. (**C**) Expression of *Cbfa2t3* and *Cyp4a14* mRNA in *Cbfa2t3*^+/+^ and *Cbfa2t3*^−/−^ mice. (**D**) Expression of *Hspa1b* and *Car5a* mRNA in *Cbfa2t3*^+/+^ and *Cbfa2t3*^−/−^ mice with and without WY14643. Each data point represents the mean ± SD for *n* = 5. * *p* < 0.05; ** *p* < 0.01; *** *p* < 0.001 vs. *Cbfa2t3*^+/+^.

GTT and ITT results showed that higher glucose tolerance and insulin sensitivity were seen in *Cbfa2t3*^−/−^ mice compared to *Cbfa2t3*^+/+^ mice. To explore the role of *Cbfa2t3* in metabolic stress in liver, WY14643 was administrated for 2 days and then the phenotype and gene expression response in *Cbfa2t3*^+/+^ mice and *Cbfa2t3*^−/−^ mice was verified. A discernable difference regarding the liver index (mg liver/g total body mass) and total body weight loss was not observed between *Cbfa2t3*^+/+^ and *Cbfa2t3*^−/−^ mice with and without WY14643 treatment (Figure 3A). Levels of triglyceride (TG) and cholesterol (CHOL) in liver were decreased by WY14643 administration, but no differences in TG and CHOL were observed between the *Cbfa2t3*^+/+^ and *Cbfa2t3*^−/−^ mice (Figure 3B). Hepatic *Cbfa2t3* mRNA expression was significantly increased in *Cbfa2t3*^+/+^ mice after WY14643 administration while no induction was observed in *Cbfa2t3*^−/−^ liver; expression of *Cyp4a14* mRNA, a classic PPARA target gene, was elevated in both *Cbfa2t3*^+/+^ and *Cbfa2t3*^−/−^ mouse lines (Figure 3C). *Hspa1b* mRNA expression was increased in *Cbfa2t3*^+/+^ livers but not in livers of *Cbfa2t3*^−/−^ mice (Figure 3D). In contrast, *Car5a* mRNA levels were repressed by WY14643 and repressed *Car5a* levels were recovered in WY14643-treated *Cbfa2t3*^−/−^ mice (Figure 3D). These data suggest that PPARA is differentially regulated by *Hspa1b* and *Car5a* and regulated by CBFA2T3.

### 3.4. Cbfa2t3 Attenuates Fasting-Induced Lipid Accumulation

**Figure 4:**
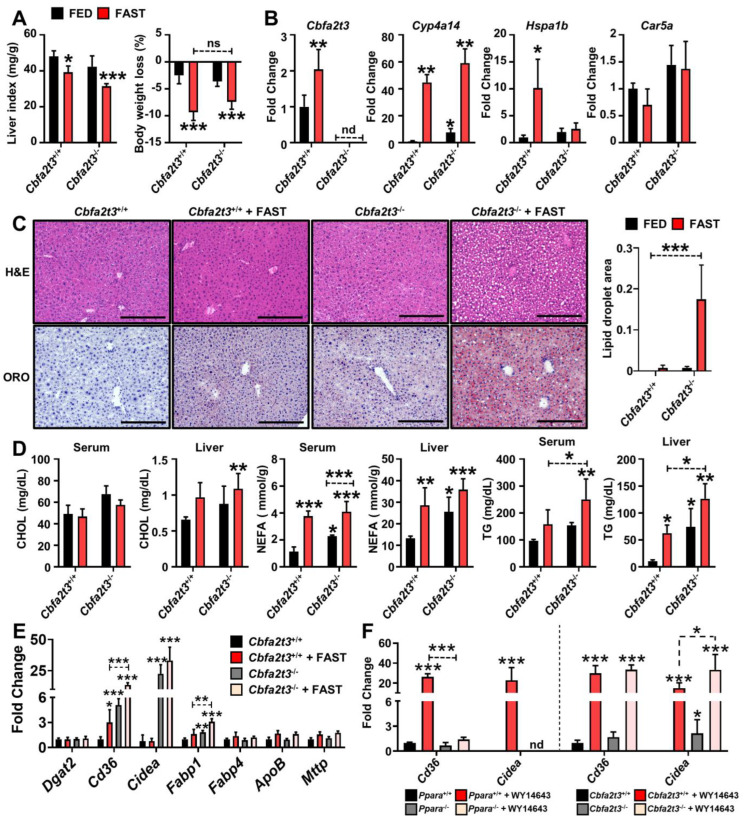
*Cbfa2t3* null causes lipid accumulation during fasting. *Cbfa2t3*^+/+^ and *Cbfa2t3*^−/−^ mice were fasted for 24 h. (**A**) Liver indexes (mg liver/g total body mass) and body weight loss after fasting. (**B**) Expression of *Cbfa2t3*, *Cyp4a14*, *Hspa1b*, and *Car5a* mRNA after 24 h of fasting. (**C**) Liver H&E and ORO staining after 24 h fasting. Scale bars represent 100 nm (200×). At least 10 cells were quantified in each image for calculating the lipid droplet area. (**D**) CHOL, NEFA and TG levels from serum and liver tissues after 24 h. (**E**) Expression of lipid accumulation-related genes after 24 h fasting. (**F**) Expression of *Cd36* and *Cidea* mRNA in liver of *Ppara*^+/+^, *Ppara*^−/−^, *Cbfa2t3*^+/+^ and *Cbfa2t3*^−/−^ mice after WY14643 administration for 2 days. Each data point represents the mean ± SD for *n* = 5. * *p* < 0.05; ** *p* < 0.01; *** *p* < 0.001 vs. *Cbfa2t3*^+/+^, vs. *Ppara*^+/+^.

PPARA activation during fasting promotes metabolic remodeling that accelerates the use of lipids as an alternate energy source with hepatosteatosis reduction [13]. To investigate whether CBFA2T3 attenuates the fasting-induced liver stress response, *Cbfa2t3*^+/+^ and *Cbfa2t3*^−/−^ mice were fasted for 24 h. A discernable difference in the liver index and body weight were not observed between fasted *Cbfa2t3*^+/+^ and *Cbfa2t3*^−/−^ mice (Figure 4A). Hepatic *Cbfa2t3* mRNA was significantly elevated in fasting *Cbfa2t3*^+/+^ mice and not detected in *Cbfa2t3*^−/−^ mice (Figure 4B). Additionally, lower expression of *Cbfa2t3* mRNA was identified following WY14643 administration (c.f., Figure 3C). *Cy4a14* is a classic PPARA target and its mRNA expression was increased in liver of fasted *Cbfa2t3*^+/+^ and *Cbfa2t3*^−/−^ mice (Figure 4B). Interestingly induction of *Cyp4a14* mRNA expression was seen in *Cbfa2t3*^−/−^ mice (Figure 4B). The fasting responses of *Hspa1b* and *Car5a* mRNA expression were similar after WY14643 treatment (Figure 4B c.f., Figure 1D).

Liver lipid droplets as revealed by H&E were widespread in fasted *Cbfa2t3*^−/−^ mice compared to *Cbfa2t3*^+/+^ mice (Figure 4C). Oil Red O staining revealed a greater lipid accumulation response to fasting liver in *Cbfa2t3*^−/−^ mice compared to *Cbfa2t3*^+/+^ mice (Figure 4C). CHOL levels were elevated in *Cbfa2t3*^−/−^ serum and liver and increased in fasted *Cbfa2t3*^−/−^ mice compared to *Cbfa2t3*^+/+^ mice (Figure 4D). NEFA levels in serum and liver were increased in *Cbfa2t3*^−/−^, while no difference between fasting *Cbfa2t3*^+/+^ and *Cbfa2t3*^−/−^ mice was observed (Figure 4D). TG levels in serum were elevated in *Cbfa2t3*^−/−^ mice. Liver TG levels were elevated in *Cbfa2t3*^−/−^, fasted *Cbfa2t3*^+/+^ and *Cbfa2t3*^−/−^ mice (Figure 4D).

Furthermore, induction of *Cd36*, *Cidea*, and *Fabp1* mRNAs were identified in 24 h fasted *Cbfa2t3*^−/−^ mice compared to *Cbfa2t3*^+/+^ mice (Figure 4E). Induction of lipid accumulation-related genes such as diacylglycerol O-acyltransferase 2 (*Dgat2*), fatty acid binding protein 4 (*Fabp4*), apolipoprotein B (*ApoB*) and microsomal triglyceride transfer protein (*Mttp*) [16] was not observed in *Cbfa2t3*^−/−^ mice compared to *Cbfa2t3*^+/+^ (Figure 4E). *Cd36* and *Cidea* mRNA expression levels were increased by WY14643 administration in the liver of *Ppara*^+/+^ and completely abolished in the liver of *Ppara*^−/−^ mice (Figure 4F). However, basal expression levels of *Cidea* mRNA were increased in *Cbfa2t3*^−/−^ mice compared to *Cbfa2t3*^+/+^ mice (Figure 4F). Interestingly, induction of *Cidea* mRNA expression was significantly elevated in *Cbfa2t3*^−/−^ liver compared to *Cbfa2t3*^+/+^ liver, suggesting *Cbfa2t3* might modulate lipid accumulation though *Cidea* regulation under metabolic stress in liver (Figure 4F). These results suggest that *Cbfa2t3* may have a modulatory role in the hepatic fasting response mediated by PPARA.

## 4. Discussion

CBFA2T3 is a member of a transcriptional corepressor family that modulates progenitor cell self-renewal, lineage commitment, and T-cell development [20]. Fusion genes with CBFA2T3–RUNX1 or GBFA2T3–GLIS2 results in an extremely aggressive leukemia phenotype [21]. In cancer studies, CBFA2T3 has anti-cancer properties in azoxymethane/dextran sulfate sodium-induced colon tumorigenesis and anti-proliferative activity in human breast cancer cells [7,11]. Pharmacological and physiological PPARA activation by WY14643 and fasting are important regulatory mechanisms of lipid and glucose homeostasis [16]. PPARA regulates metabolic remodeling, inflammation, and hepatocyte proliferation by modulating various target gene’s expression [15]. Prolonged PPARA activation promotes hepatocyte proliferation by activation of keratin 23, a PPARA-dependent MYC-amplified oncogene [15]. PPARA activation promotes TXNIP antisense lncRNA Gm15441 activation, which attenuates fasting-induced metabolic stress through TXNIP-mediated NLRP3 inflammasome pathway suppression in liver [16]. LncRNA G23Rik is a PPARA-sensitive gene that modulates fasting-induced metabolic stress in liver [17].

However, the modulation mechanism of metabolic stress by PPARA is largely unknown. RNA-seq analysis identified that PPARA activation promoted *Cbfa2t3* induction in mouse liver and induction of *Cbfa2t3* was completely abolished in *Ppara*^−/−^ mouse liver [16]. Previous studies showed that PPARA target genes play a modulatory role in metabolic remodeling [16,17]. In the current study, the role of CBFA2T3 in metabolic stress modulation in mouse liver was investigated. Basal *Cbfa2t3* mRNA expression is very low in liver but abundant in lung, heart, and BAT. However, hepatic *Cbfa2t3* mRNA expression levels are highly elevated after WY14643 treatment of wild-type mice but not *Ppara*^−/−^ mice, demonstrating that *Cbfa2t3* is a PPARA target gene. Gene expression monitoring results revealed that *Cbfa2t3* mRNA expression is a response at an early time point and is robustly elevated after 6 h of PPARA activation. These results indicate that hepatic *Cbfa2t3* is PPARA sensitive and suggest that it is involved in cell proliferation and fatty acid metabolism and transport, both of which are under the control of PPARA in mouse liver.

PPARA activates a subset of its target genes in mouse liver [15]. HSPA1B is a typical stress-induced chaperone protein that is regulated by miR-15a through directly binding to its 3′-UTR [22]. Recently, single-cell profiling analysis of human CD127^+^ innate lymphoid cells from human patients with hepatocellular carcinoma (HCC) revealed that HSPA1A and HSPA1B expression were highly activated in late-state HCC, suggesting HSPA1B is important in tumor microenvironment remodeling [23]. Single-cell analysis of the GSE129516 dataset and expression profiling GSE184019 dataset analysis revealed that HSPA1B expression is elevated in non-alcoholic steatohepatitis (NASH) [24]. Wang et al. suggested that HSPA1B could be a diagnostic and prognostic biomarker and a potential therapeutic target for NASH [24]. However, the molecular mechanism by which HSPA1B modulates remodeling is still not clear.

CAR5A is important for ureagenesis and gluconeogenesis, and *Car5a*-null mice are smaller than *Car5a* wild-type littermates [25]. However, sodium and potassium citrate-containing water administration rescue the offspring in the expected numbers [25]. Fasting glucose levels were normal, but blood ammonia concentration was significantly elevated in *Car5a*-null mice, suggesting that Car5a is highly expressed in liver and modulates ammonia detoxification [25]. Car5A/B double-knockout mice revealed a phenotype of hyperammonemia, smaller than littermates, a poor survival rate and lower fasting glucose levels compared to *Car5a*-null mice [25]. Recently, a study showed that *Car5a* has a predominant role in ammonia detoxification, whereas the roles of *Car5b* in ureagenesis and gluconeogenesis are essential only in the absence of *Car5a* [26]. In a clinical study, metabolic disorders in children associated with *Car5a* were more common than other metabolic disorders and responded well to treatment with N-carbamyl-l-glutamate [26]. However, the regulatory mechanism of *Car5a* on metabolic remodeling is largely unknown.

In this study, hepatic *Hspa1b* mRNA expression was increased by WY14643 and abolished in *Cbfa2t3*^−/−^ mice. Gene expression monitoring results showed that maximum *Hspa1b* mRNA expression was at 12 h after WY14643 administration, the same expression pattern as *Cbfa2t3* mRNA expression. A previous study revealed that PPARA directly activates MYC expression and MYC amplifies PPARA target gene expression [15]. Gene expression profiling analysis from β-catenin and YAP-induced hepatoblastoma mouse models identified that a high expression level of *Hspab1* was seen in the liver of *Myc*^+/+^ mice but not *Myc*^−/−^ mice [27]. These results indicate that multiple mechanisms, such as PPARA and MYC, are involved in *Cbfa2t3* mRNA expression in liver. Hepatic *Car5a* mRNA expression was repressed by WY14643 and rescued in *Cbfa2t3*^−/−^ mice. However, *Car5a* mRNA expression was rapidly suppressed after WY14643 administration, and the maximum suppression time point was 12 h.

Interestingly, the expression pattern of *Car5a* mRNA induced by WY14643 was opposite to that of *Cbfa2t3* mRNA expression. PPARA ChIP-sequence analysis identified potential PPARA binding sites within the *Car5a* transcriptional start site, suggesting PPARA may directly regulate *Car5a* gene expression [28]. However, no significant expression pattern was seen in the fasting liver of *Cbfa2t3*^+/+^ mice and *Cbfa2t3*^−/−^ mice, suggesting multiple mechanisms may be involved in *Car5a* expression in liver. These results suggest that PPARA may have a repressive role on *Car5a* during metabolic stress in liver. However, the mechanism of gene repression by PPARA was recently uncovered. Taken together, these data suggest that *Hspa1b* and *Car5a* may be PPARA-sensitive and CBFA2T3-regulated genes that modulate stress and homeostasis in mouse liver.

PPARA directly modulates the metabolic stress response, hepatosteatosis, and lipid accumulation through direct activation of PPARA target genes such as *Gm15441* and *G23Rik* [16,17]. To verify the modulatory role of *Cbfa2t3* on metabolic stress, physiological PPARA activation by a fasting model was used. Histology analysis identified that lipid droplets appeared in the fasting liver of *Cbfa2t3*^+/+^ mice but were more widely spread in the fasting liver of *Cbfa2t3*^−/−^ mice. Lipid accumulation was seen in the fasting liver of *Cbfa2t3*^−/−^ mice compared to that of *Cbfa2t3*^+/+^ mice by ORO staining. The levels of liver CHOL, NEFA, and TG were significantly induced by fasting in *Cbfa2t3*^−/−^ mice compared to *Cbfa2t3*^+/+^ mice. Serum NEFA and TG levels were significantly elevated in *Cbfa2t3*^−/−^ mice compared to *Cbfa2t3*^+/+^ mice. Interestingly, NEFA and TG levels in serum and liver were significantly elevated in *Cbfa2t3*^−/−^ mice compared to *Cbfa2t3*^+/+^ mice. These results suggest that *Cbfa2t3* has a regulatory role in metabolic stress. qRT-PCR analysis results showed that mRNA expression of lipid accumulation-related genes such as *Cd36*, *Cidea*, and *Fabp1* were induced in fasting *Cbfa2t3*^−/−^ liver compared to *Cbfa2t3*^+/+^ liver. *Cd36*, *Cidea*, and *Fabp1* are considered PPARA-dependent genes that are highly expressed under metabolic stress in liver and could be potential therapeutic targets for nonalcoholic fatty liver disease [29,30,31]. Especially, basal expression levels of *Cbfa2t3* mRNA were significantly elevated in *Cbfa2t3*^−/−^ mice compared to *Cbfa2t3*^+/+^ mice, and much higher induction was seen in *Cbfa2t3*^−/−^ liver compared to *Cbfa2t3*^+/+^ liver after WY14643 administration. Excessive expression of *Cidea* promoted lipid accumulation, lipid droplets, and hepatic steatosis in mice and humans [32]. High fat diet-induced lipid accumulation and hepatic steatosis were reduced in *Cidea*^−/−^ liver compared to *Cidea*^+/+^ liver [32]. Triglyceride levels in milk collected from female *Cidea*^−/−^ mice were reduced around 70% compared to milk from female *Cidea*^+/+^ mice [33]. *Cidea* induction by lipopolysaccharide promoted lipid accumulation and liver steatosis in mouse liver and human hepatocytes [34]. Thus, *Cbfa2t3* regulates *Cidea* expression, which may contribute to lipid accumulation and hepatosteatosis in *Cbfa2t3*^−/−^ liver. These results indicate that *Cbfa2t3* is a PPARA-dependent gene that modulates lipid accumulation under metabolic stress conditions.

In conclusion, the PPARA-dependent hepatic CBFA2T3 protein is a metabolic modulator that is induced by PPARA induction in mouse liver. Lipid accumulation and lipid droplets were observed in fasting liver of *Cbfa2t3*^−/−^ mice but not *Cbfa2t3*^+/+^ liver. TG, CHOL, and NEFA levels in liver in fasting *Cbfa2t3*^−/−^ mice were significantly increased with induction of transcription of *Cd36*, *Cidea*, and *Fabp1*, which encode lipid metabolism-modulated proteins. Especially, basal expression levels of hepatic *Cidea* mRNA were elevated in *Cbfa2t3*^−/−^ compared to *Cbfa2t3*^+/+^ mice and highly elevated in *Cbfa2t3*^−/−^ mice after WY14643 administration, suggesting *Cbfa2t3* may modulate lipid metabolism through regulation of *Cidea*. Taken together, these results suggest that CBFA2T3 can prevent the fasting-induced liver stress response and represents a novel therapeutic target against metabolic stress modulators.

## Figures and Tables

**Table 1:** qRT-PCR primer sequences.

Name	Sequence (5′ → 3′)
ApoB F	GGTGTATGGCTTCAACCCTGA
ApoB R	GCTTGAGTTCGTACCTGGACA
Car5a F	GCAAACTTCGCTCGTCCTTC
Car5a R	TTCCGGTCTGCTCTGCCTAT
Cd36 F	GATTAATGGCACAGACGCAGC
Cd36 R	CAGATCCGAACACAGCGTAGA
Cyp4a14 F	CCTGACTTTCTTTCGCCTGC
Cyp4a14 R	TGATCACTCCATCTGTGTGCT
Dgat2 F	GGTCTGCAGCCAGAGAAGAG
Dgat2 R	TCCAGGTATGAGGAGTCTTCC
Fabp1 F	AGTCAAGGCAGTCGTCAAGC
Fabp1 R	ATGTCGCCCAATGTCATGGT
Fabp4 F	CATAACCCTAGATGGCGGGG
Fabp4 R	CGCCTTTCATAACACATTCCACC
Gapdh F	GACTTCAACAGCAACTCCCAC
Gapdh R	TCCACCACCCTGTTGCTGTA
Hspa1b F	CGAGGAGGTGGATTAGAGGC
Hspa1b R	TGCCCAAGCAGCTATCAAGT

## Data Availability

The original contributions presented in the study are included in the article.
